# Factors associated with symptom development in bilateral hip dysplasia with comparable acetabular coverage

**DOI:** 10.1007/s00264-026-06917-w

**Published:** 2026-06-24

**Authors:** Hiroto Funahashi, Yusuke Osawa, Hiroaki Ido, Takamune Asamoto, Yasuhiko Takegami, Shiro Imagama

**Affiliations:** Department of Orthopedic Surgery, Nagoya University, Nagoya, Japan

**Keywords:** Hip dysplasia, Lateralization, Femoral neck anteversion, Acetabular sector angle, Centre–edge angle

## Abstract

**Purpose:**

This study aimed to identify morphological differences between the symptomatic and asymptomatic hips in patients with bilateral hip dysplasia (HD).

**Methods:**

From 109 patients who underwent eccentric rotational acetabular osteotomy (ERAO) between 2017 and 2022, 33 patients were selected as the First Cohort. These patients had undergone ERAO on the symptomatic side, while the contralateral side remained completely asymptomatic for ≥ three years postoperatively. Among them, 18 patients with matching lateral centre-edge angles (CEA) on both sides were selected as the Second Cohort. The acetabular sector angles (ASAs) were measured three-dimensionally. Femoral neck anteversion (FNA) and femoral head lateralization were also assessed. Morphological comparisons between the symptomatic and asymptomatic sides were conducted using paired t-tests in both cohorts. In the Second Cohort, conditional logistic regression analysis was used to identify factors associated with the onset of hip pain.

**Results:**

In the First Cohort, the symptomatic side exhibited lower ASAs across the superior-to-anterior acetabulum than the asymptomatic side, whereas no significant differences in ASAs were observed in the Second Cohort. By contrast, both cohorts exhibited greater FNA and lateralization on the symptomatic side. Conditional logistic regression analysis in the Second Cohort identified increased FNA (odds ratio: 1.3) and lateralization (odds ratio: 4.6) as factors associated with hip pain (all *p* < 0.05).

**Conclusion:**

When the CEA is comparable on both sides in patients with bilateral HD, morphological features, such as increased FNA and femoral head lateralization, may be more indicative of the symptomatic hip than acetabular parameters.

## Introduction

Hip dysplasia (HD) is a prevalent disorder characterised by insufficient acetabular coverage, resulting in elevated intra-articular contact pressure [1], labral tears, hip pain [2, 3], and functional limitation. These factors contribute to the eventual development of secondary osteoarthritis (OA) [4, 5]. The morphology of the dysplastic hip varies considerably, with diverse three-dimensional patterns of acetabular deficiency [6] and abnormal obliquity of the acetabular roof [7]. The center–edge angle (CEA) is a key parameter used to evaluate HD severity; lower CEA values are associated with increased pain risk and OA progression [8]. However, in some patients with bilateral HD and comparable CEAs in both hips, only one hip becomes symptomatic, while the contralateral side remains asymptomatic. In such instances, morphological differences beyond acetabular coverage may underlie symptom development, although the specific contributing factors remain unclear.

Previous investigations into the determinants of hip pain and OA progression in HD have identified both patient-related factors such as body mass index and activity level [4, 9] and morphological characteristics of the hip joint [8]. Several morphological parameters have been evaluated as possible contributors to pain and progression, including anterior acetabular coverage, pelvic tilt [10], and femoral morphology [7, 11], in addition to the CEA. However, substantial inter-individual variation in body habitus and skeletal morphology has made it challenging to delineate clear associations between specific morphological features and the onset of symptoms or disease progression. Understanding which morphological features are associated with the onset of pain in HD may provide clinically useful information for predicting future symptom development, counselling patients, and planning periacetabular osteotomy. Comparing three-dimensional bony morphology between the symptomatic and asymptomatic hips within the same patient may help identify morphological features associated with pain. This within-patient comparative approach offers several advantages: (1) it controls for patient-specific variables, such as age, height, and weight; (2) the bilateral hips of the same individual are expected to have similar baseline morphology, allowing for the detection of subtle side-to-side differences; and (3) by minimising inter-individual variability and reducing confounding, it increases the ability to detect morphological differences with a smaller sample size, thereby improving statistical efficiency.

We hypothesized that not only superior acetabular morphology but also anteroposterior acetabular morphology and femoral morphology may underlie symptom development. Accordingly, to examine within-patient side-to-side differences in three-dimensional hip morphology, we addressed three clinical questions: (1) Are there differences in acetabular morphology between the symptomatic and asymptomatic hips in patients with bilateral HD? (2) Are there differences in femoral morphology and femoral head lateralization between the symptomatic and asymptomatic hips? (3) What hip morphological differences are present between symptomatic and asymptomatic HD hips with comparable CEA?

## Methods

### Study design and setting

This retrospective observational study was conducted at a single institution and received approval from the institutional review board (approval number: 2020–272). This study complies with the Declaration of Helsinki. Informed consent was obtained from all participants included in the study. A total of 109 patients who underwent eccentric rotational acetabular osteotomy (ERAO) for HD between January 2017 and March 2022 were initially identified. ERAO, a form of periacetabular osteotomy, involves a spherical cut of the acetabulum followed by lateral rotation, and has been shown to provide favourable postoperative outcomes [12, 13].

### Participants/study subjects

**Fig. 1:**
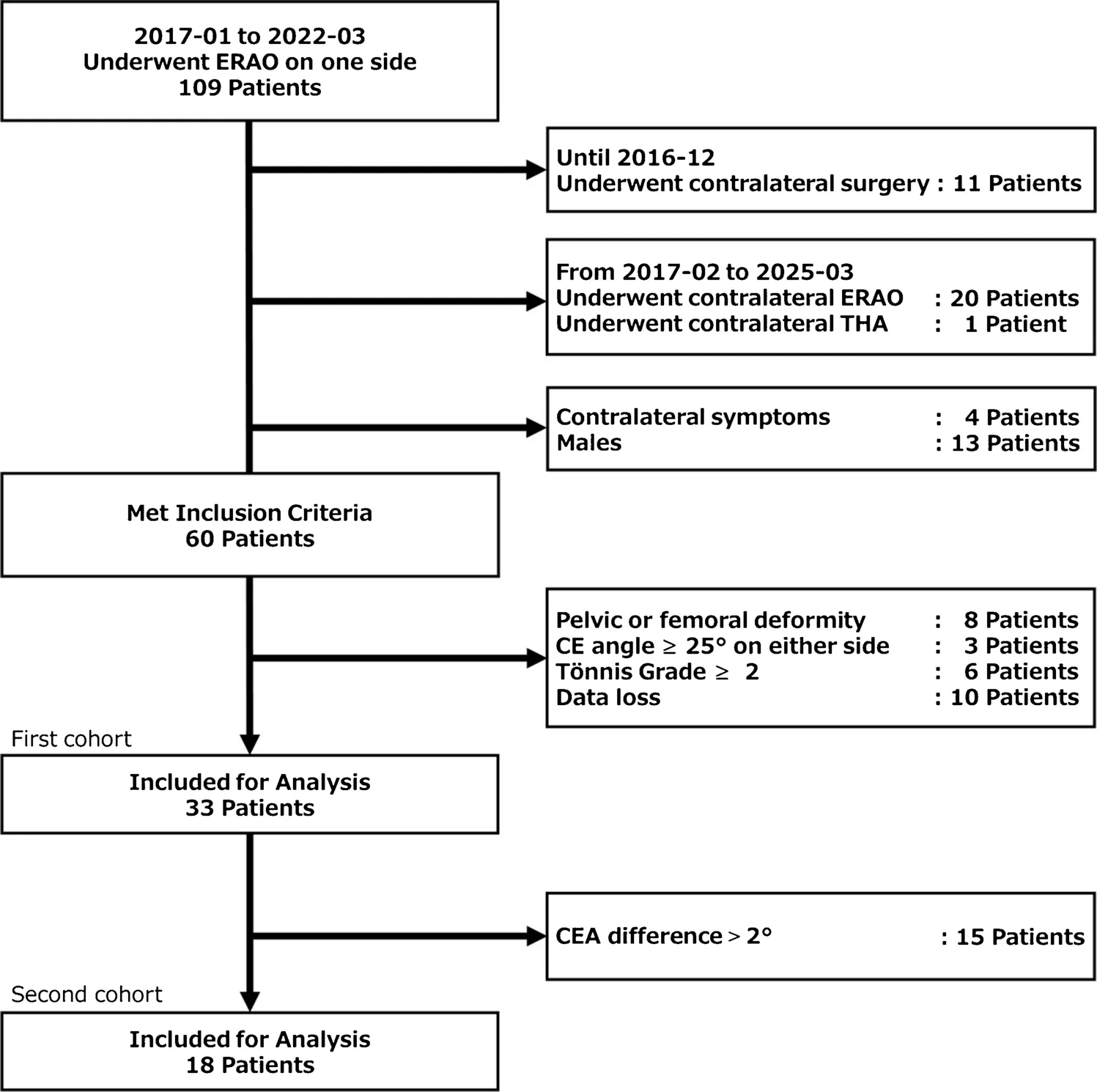
STROBE flowchart of this study. ERAO = eccentric rotational acetabular osteotomy, CEA = centre–edge angle, STROBE = Strengthening the Reporting of Observational Studies in Epidemiology

Among the 109 patients, 11 were excluded due to prior hip surgery—including ERAO—on the affected side before the index procedure. As of March 2025, further exclusions were applied: patients who had undergone ERAO or total hip arthroplasty on the contralateral side, those who developed contralateral symptoms (including pain), and all male patients. This resulted in the inclusion of 60 female patients who had received ERAO on the symptomatic side due to pain, while the contralateral hip remained asymptomatic for ≥ three years postoperatively. Patients with a contralateral center–edge angle (CEA) > 25°, marked hip deformity, advanced OA (Tönnis grade > 2), or incomplete data were excluded. The remaining 33 patients comprised the First Cohort. As a subset analysis of the First Cohort, 18 patients with a ≤ 2° difference in CEA between the symptomatic and asymptomatic sides (measured on anteroposterior radiographs) were defined as the Second Cohort (Fig. 1; Table 1).

**Table 1:** Patient demographic and radiological data

Parameter	First Cohort	Second Cohort
Patients, *n*	33	18
Median age, years (IQR)	30 (13 to 52)	20 (13 to 51)
Median body mass index, kg/m[2] (IQR)	21 (16 to 33)	21 (18 to 33)
Laterality of the symptomatic side, n (%)	20 (61)/13 (39)	10 (56)/8 (44)
Tönnis grade of the affected side (1/2), n (%)	17 (52)/16 (48)	8 (44)/10 (56)
Mean CEA (symptomatic/asymptomatic), º SD	17 ± 5/15 ± 6	17 ± 3/15 ± 4

*IQR* interquartile range, *CEA* center–edge angle, *SD* standard deviation

### Evaluations

**Fig. 2:**
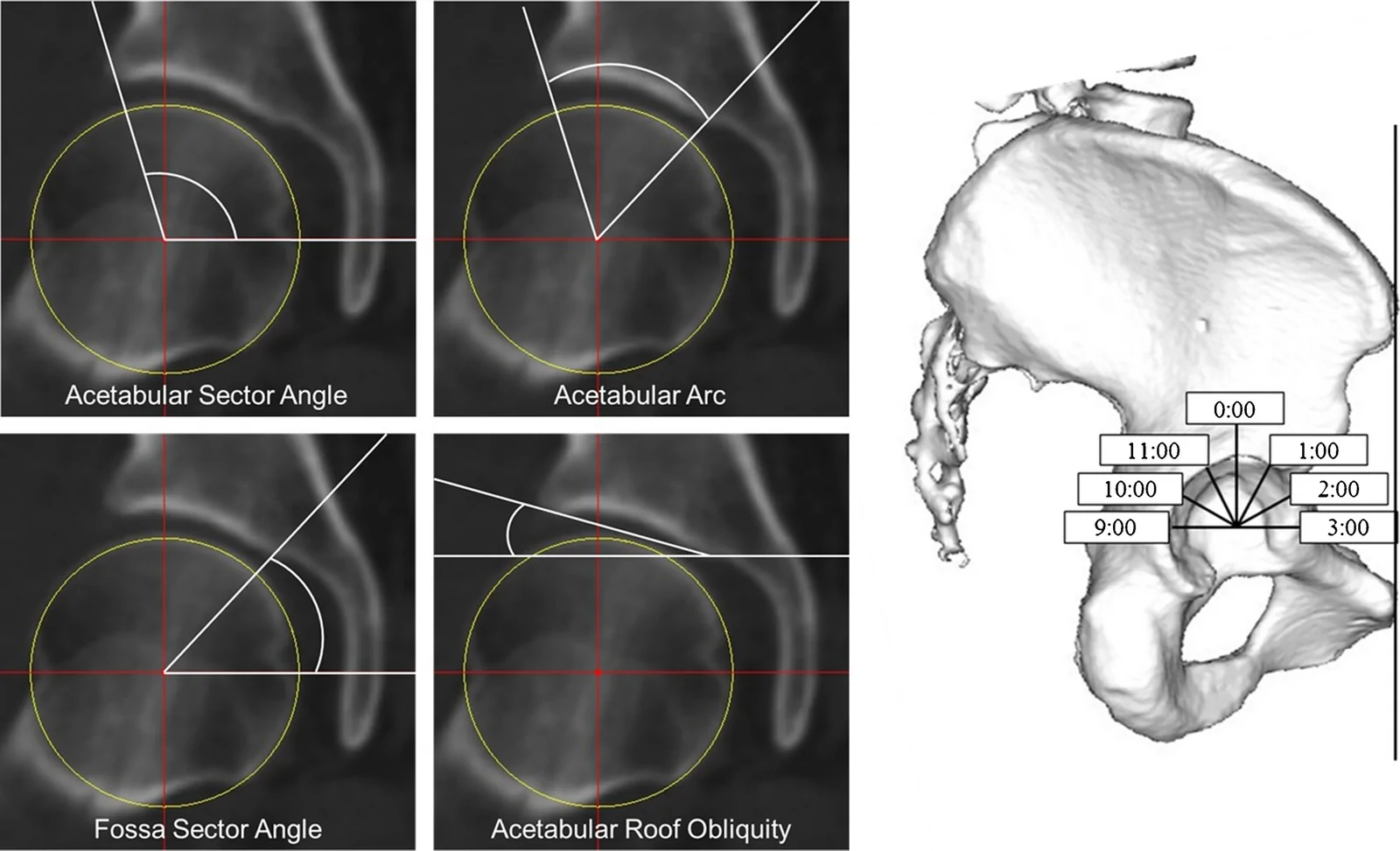
Pelvic morphology assessed using the anterior pelvic plane (APP) coordinate system. All diagrams show measurements obtained at the 12 o’clock orientation. Similar evaluations were performed radially from the centre of the femoral head

**Fig. 3:**
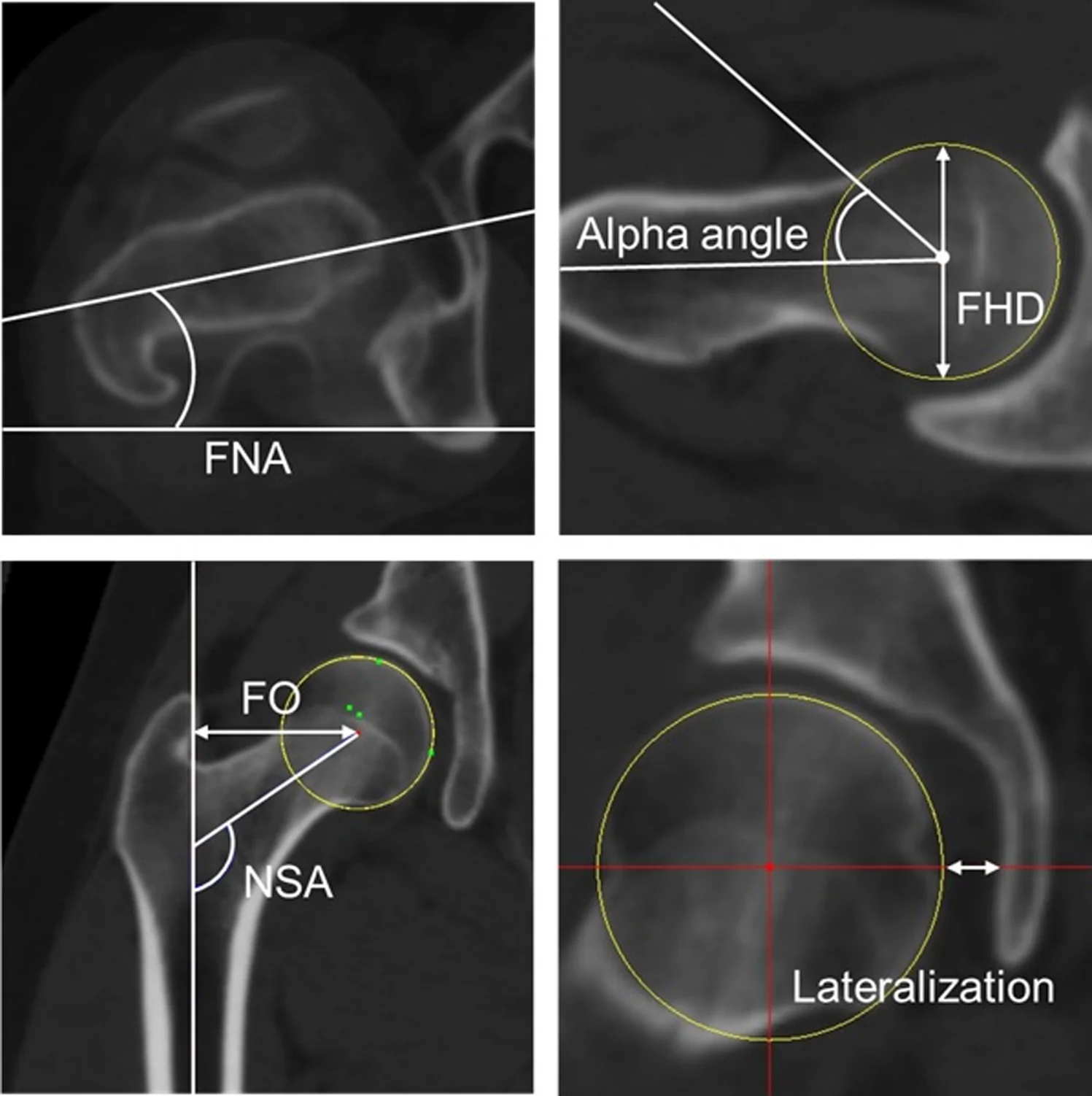
Femoral morphology assessment. FNA, alpha angle, and NSA were defined as the angles formed by the following pairs of lines: FNA: the femoral neck axis and posterior condylar axis. Alpha angle: the femoral neck axis and a line connecting the femoral head centre to the point where the head-neck junction deviates from sphericity. NSA: the femoral neck axis and femoral shaft axis. FO was defined as the perpendicular distance from the centre of the femoral head to the femoral shaft axis. Lateralization was defined as the distance from the centre of the femoral head to the acetabular fossa, measured parallel to the X-axis in the anterior pelvic plane coordinate system. FNA = femoral neck anteversion, NSA = neck-shaft angle, FO = femoral offset

All patients had undergone preoperative CT scanning of the pelvis to the knee using a Toshiba Aquilion scanner (Toshiba Medical), with 1 mm slice thickness, 120 kV, and 320 mA. Data were processed using ZedHip version 17.0.0 (Lexi Co., Ltd.). Pelvic models were aligned to the anterior pelvic plane (APP) coordinate system. Acetabular sector angles (ASAs) were measured using a clock-face reference, with the cranial direction defined as 0 o’clock in the anterior APP view, and measured radially toward the anterior pelvis. Additional assessments included the acetabular arc (AA), fossa sector angle (FSA), and acetabular roof obliquity (ARO), all based on the same coordinate system to ensure comprehensive evaluation of acetabular morphology (Fig. 2). Femoral morphology was assessed using multiple parameters, including femoral neck anteversion (FNA) [14], neck-shaft angle (NSA), femoral offset (FO), femoral head diameter, and alpha angle [15–20]. Based on established radiographic methods [10], lateralization was evaluated using CT measurements of the distance from the medial edge of the femoral head to the inner cortex of the acetabular fossa (Fig. 3).

### Statistical analysis

All measurements were independently performed twice by the first and third authors, with a minimum interval of one month between sessions. CT-based assessments demonstrated excellent intra- and interobserver reliability: intraobserver intraclass correlation coefficients (ICCs) ranged from 0.78 to 0.91, and interobserver ICCs ranged from 0.83 to 0.94. Sample size was calculated based on the primary outcome. Preliminary data from the present study on the side-to-side difference in femoral head lateralization showed a mean difference of 1.2 mm with a standard deviation of 1.6 mm. Using a paired t-test with a significance level of 0.05 and power of 80%, the required sample size was estimated at 16, confirming the adequacy of the current sample.

In both cohorts, paired t-tests were used to compare hip morphology between the symptomatic and asymptomatic sides. Normality was verified using the Kolmogorov–Smirnov test, which confirmed normal distribution of all variables. Analyses were performed in the following sequence: (1) acetabular parameters were compared between sides in the First Cohort and then in the Second Cohort; (2) femoral parameters, including femoral head lateralization, were compared between sides in the First Cohort and then in the Second Cohort; and (3) in the Second Cohort, parameters showing significant side-to-side differences were entered into a conditional logistic regression model to identify morphological factors associated with hip pain onset. Statistical significance was set at *p* < 0.05. All analyses were conducted using EZR version 1.41 (64-bit) (Saitama Medical Center, Jichi Medical University), a graphical user interface for R [21].

## Results

**Fig. 4:**
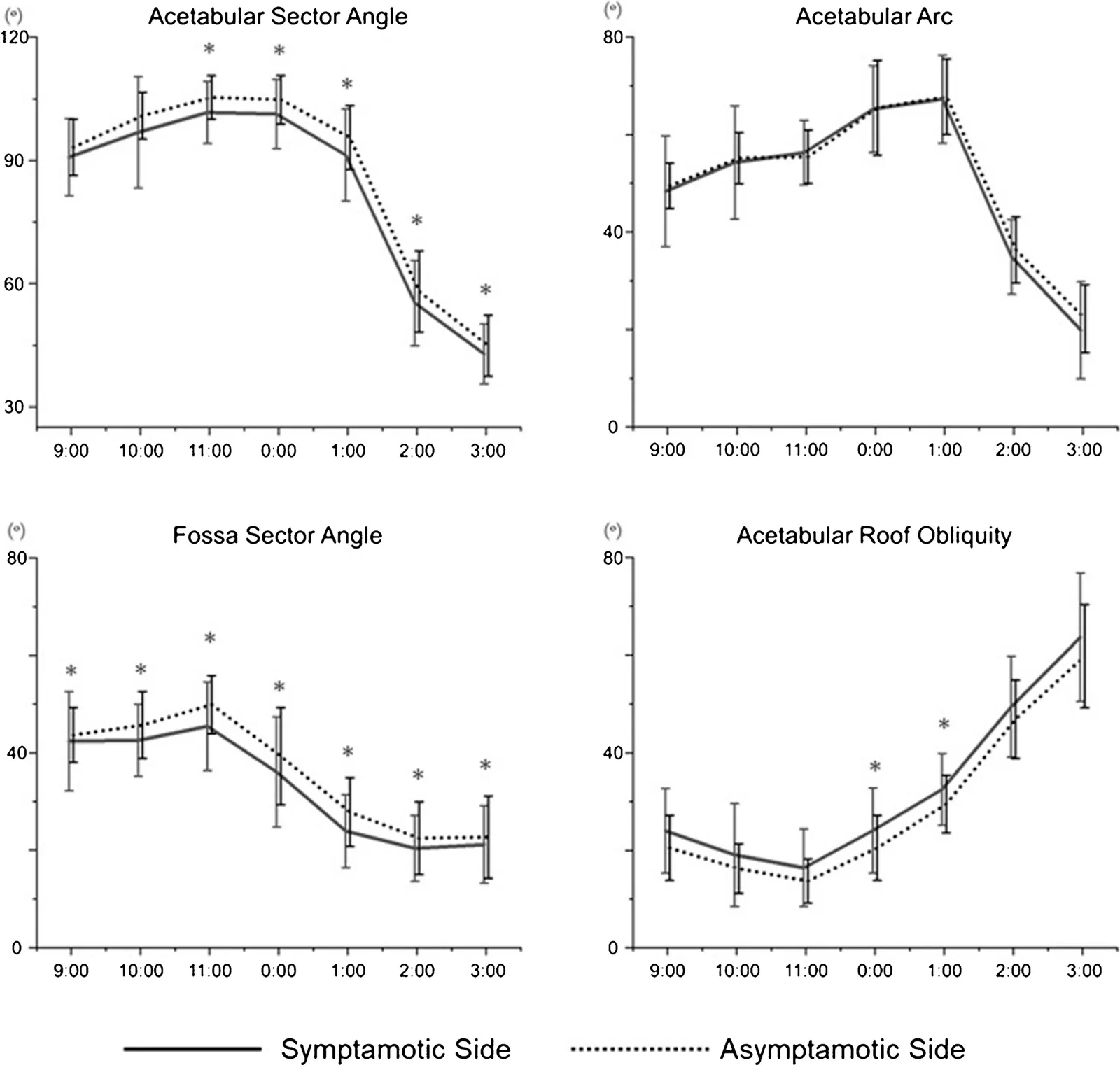
Acetabular morphology assessment in the first cohort. * < 0.05

**Fig. 5:**
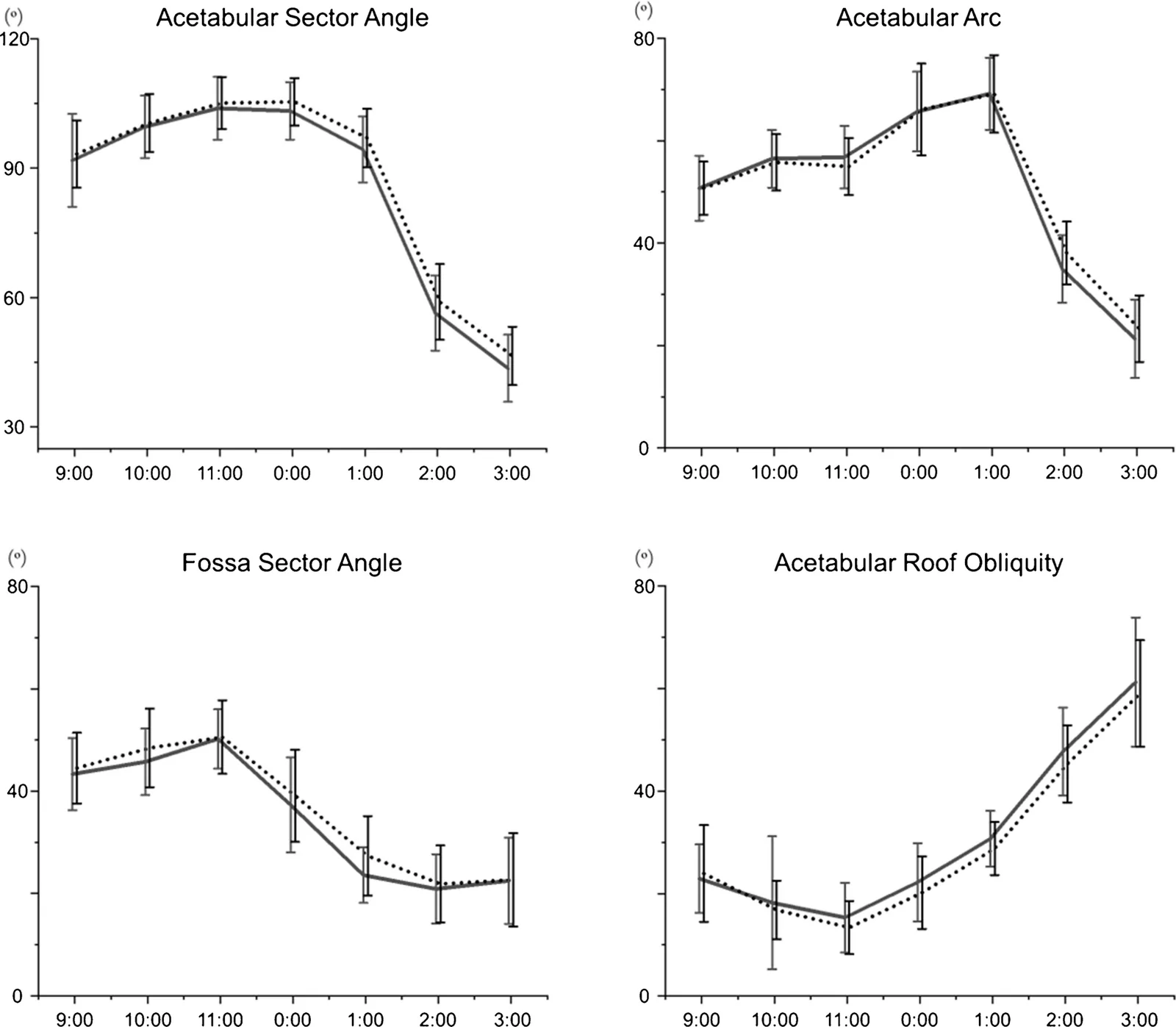
Acetabular morphology assessment in the second cohort. * < 0.05

In the First Cohort, the ASA was lower on the affected side from 11:00 to 3:00 (all *p* < 0.05). The FSA was consistently reduced on the affected side across all directions (all *p* < 0.05). ARO was higher at 9:00, 12:00, and 1:00 on the affected side (all *p* < 0.05). No significant difference in AA was observed between sides (Fig. 4).　Analysis of the direction with the lowest p-value for each parameter (asymptomatic side – symptomatic side) showed that ΔASA at 0:00 (median: 2°, interquartile range [IQR]: –1 to 6) was greater on the affected side in 30.3% of cases; ΔARO at 1:00 (median: −2°, IQR: –8 to 1) was lower in 33.3%; and ΔFSA at 11:00 (median: 4°, IQR: 0 to 11) was higher in 24.2%. In the Second Cohort, where CE angles were matched bilaterally, no significant differences in ASA, AA, FSA, and ARO were found between the symptomatic and asymptomatic sides (Fig. 5).

In the First Cohort, comparison of femoral morphology revealed that FNA was greater on the affected side (23 ± 10° vs. 21 ± 9°, *p* = 0.027), while FO was reduced (33 ± 4 mm vs. 35 ± 5 mm, *p* = 0.028). Lateralization was also greater on the affected side (10 ± 2 mm vs. 9 ± 1 mm, *p* < 0.001) (Table 2). Assessment of side-to-side differences (asymptomatic side – symptomatic side) showed that ΔFNA (median: −4.3°, IQR: −9 to 0) was higher on the affected side in 18.2% of cases; ΔFO (median: 1 mm, IQR: 0 to 3) was lower in 30.3%; and ΔLateralization (median: −1 mm, IQR: −2 to 0) was higher in 15.2%. In the Second Cohort, FNA remained greater on the affected side (24 ± 11° vs. 20 ± 11°, *p* = 0.033), and lateralization was similarly increased (9 ± 2 mm vs. 8 ± 2 mm, *p* = 0.028) (Table 3).

**Table 2:** Analysis of the first cohort: comparison of femoral morphology and lateralization

	symptomatic side	asymptomatic side	*P*-value
Femoral neck anteversion (°)	23 ± 10	21 ± 9	0.027^*^
Alpha angle (°)	41 ± 5	41 ± 5	> 0.99
Femoral head diameter (mm)	21 ± 2	21 ± 1	0.87
Femoral offset (mm)	33 ± 4	35 ± 5	0.028^*^
Neck shaft angle (°)	131 ± 6	127 ± 10	0.05
Latelarization (mm)	10 ± 2	9 ± 1	< 0.001^*^

^*^P-value < 0.05

**Table 3:** Analysis of the second cohort: comparison of femoral morphology and lateralization

	symptomatic side	asymptomatic side	*P*-value
Femoral neck anteversion (°)	24 ± 11	20 ± 11	0.033^*^
Alpha angle (°)	41 ± 5	41 ± 5	0.70
Femoral head diameter (mm)	21 ± 2	21 ± 1	0.83
Femoral offset (mm)	36 ± 4	36 ± 5	0.55
Neck shaft angle (°)	130 ± 7	125 ± 11	0.13
Latelarization (mm)	9 ± 3	8 ± 2	0.028^*^

^*^P-value < 0.05

Conditional logistic regression analysis of morphological differences in the Second Cohort showed that both FNA (odds ratio: 1.33, 95% confidence interval [CI]: 1.01–1.75, p = 0.041) and lateralization (odds ratio: 4.64, 95% CI: 1.08–20.00, p = 0.040) were associated with the onset of hip pain.

## Discussion

Although bilateral HD can lead to pain in both hips in some patients [22, 23], others remain asymptomatic on the contralateral side despite apparently comparable acetabular coverage. We hypothesized that, in addition to superior acetabular morphology, anterior–posterior acetabular morphology and femoral morphology may contribute to the development of symptoms. To explore this, we analyzed patients with bilateral HD who underwent ERAO on the symptomatic side due to significant pain, while the contralateral side remained entirely asymptomatic. Comparative morphological assessment revealed that the symptomatic side demonstrated reduced ASA from superior to anterior directions, increased ARO in the anterosuperior region, greater FNA, decreased FO, and more pronounced lateralization of the femoral head. Additionally, among patients with comparable CEA bilaterally, increased FNA and femoral head lateralization were identified as factors associated with the onset of hip pain.

Characteristic features of HD, including diminished acetabular coverage and increased ARO previously reported in the literature [7], were more prominent on the symptomatic side in the First Cohort. Specifically, ASA was lower in the superior to anterior regions, ARO was higher superiorly, and FSA was reduced circumferentially on the symptomatic side. Nevertheless, when assessing the distribution of side-to-side differences, 30.3% of patients exhibited greater ASA (0:00), 33.3% showed lower ARO (1:00), and 24.2% demonstrated greater FSA (11:00) on the symptomatic side. These results suggest that acetabular morphology associated with HD alone do not necessarily predict the onset of symptoms. In the Second Cohort, in which lateral CEA was matched bilaterally, no significant differences in ASA, AA, FSA, and ARO were observed between the symptomatic and asymptomatic sides. Although previous studies have suggested that appropriate anterior acetabular coverage is associated with progression of OA [14], no significant differences in anterior coverage were found between the symptomatic and asymptomatic sides in the Second Cohort. This discrepancy with the results of prior studies may be due to the within-patient comparison in our study, where both hips likely shared similar acetabular morphology. These findings suggest that in cases without substantial side-to-side differences in lateral CEA, acetabular morphology alone is not sufficient to distinguish the symptomatic side.

Comparison of femoral morphology and femoral head lateralization between the symptomatic and asymptomatic sides revealed greater FNA and more marked femoral head lateralization on the symptomatic side in both cohorts. Previous research has shown that FNA increases with the severity of HD [24, 25], and studies by Kohno et al. and Tönnis et al. have linked increased FNA to earlier hip pain onset [7, 11]. Additionally, lateralization is considered a key morphological feature of hip instability [26]. Tachibana et al. reported significantly greater lateral displacement of the femoral head in patients with HD compared to in healthy individuals [10]. These findings suggest that FNA and lateralization are critical morphological contributors to hip pain, independent of acetabular coverage. Even when acetabular parameters—such as CEA—are comparable between sides, femoral factors—such as increased FNA and lateralization—may influence symptom development. Although clinical focus in HD has traditionally been centered on acetabular morphology, these findings underscore the need for comprehensive evaluation of femoral morphology to better understand the underlying pathology.

Analysis was conducted using the Second Cohort, which included only cases with matched CEA. In this group, acetabular factors lost statistical significance, whereas FNA (odds ratio: 1.3) and femoral head lateralization (odds ratio: 4.6) emerged as factors associated with the onset of hip pain. Previous studies employing finite element analysis have demonstrated that increased FNA leads to elevated contact pressure within the hip joint [27]. Moreover, greater lateralization is believed to increase joint contact pressure at the acetabular rim, thereby contributing to cartilage and labral damage [28] Collectively, these findings suggest that in patients with bilateral HD and comparable CEA bilaterally but unilateral symptoms, surgeons should consider the potential role of increased FNA and femoral head lateralization in the development of pain. Furthermore, the present findings indicate that increased FNA and femoral head lateralization may be associated with the onset of hip pain not only in patients with bilateral HD presenting with unilateral symptoms but also in a broader clinical context. Although the present study focused on a specific patient population, these results underscore the importance of evaluating femoral morphology in addition to acetabular coverage during routine clinical assessment. Further prospective studies are warranted to determine whether these associations hold true across different patient groups and stages of the disease.

### Limitations

First, the follow-up duration for the asymptomatic hip may be relatively short. In this study, the contralateral hip remained entirely asymptomatic for ≥ three years following ERAO on the symptomatic side. Nonetheless, the asymptomatic side may develop symptoms in the future. Despite this, morphological comparisons between symptomatic and asymptomatic sides at the time of evaluation provide clinically meaningful insights. It remains important to monitor whether symptoms develop later and whether femoral head lateralization is present at that time. Second, the study included only female patients of Asian descent; male patients were excluded. This selection was based on existing evidence indicating significant differences in acetabular and femoral morphology between males and females and across ethnic groups [29]. However, as HD is more prevalent in female patients, the study design reflects the most common clinical presentation. Finally, physical characteristics such as sex, height, weight, and pelvic tilt—potential contributors to symptom onset—were not considered. As the study employed within-subject comparison, these patient-specific factors were inherently controlled. While recent evidence suggests that body mass index can influence the progression of OA [8, 30], these variables were not assessed here. Furthermore, because acetabular morphology varies by sex, this study included only female patients [31]. However, this design may have allowed for a more accurate identification of the relationship between bone morphology and pain onset by minimising confounding from physical attributes.

## Conclusions

In summary, we examined morphological factors associated with hip pain by comparing the symptomatic and asymptomatic sides in patients with bilateral HD. Among those with comparable acetabular coverage bilaterally, increased FNA and femoral head lateralization were identified as risk factors for symptom development. Further longitudinal studies are required to elucidate the relationship between hip morphology and the onset of hip pain across different patient populations.

## Data Availability

No datasets were generated or analysed during the current study.
